# Anti-inflammatory and immunomodulatory effects of the extracellular vesicles derived from human umbilical cord mesenchymal stem cells on osteoarthritis via M2 macrophages

**DOI:** 10.1186/s12951-021-01236-1

**Published:** 2022-01-20

**Authors:** Kanglu Li, Guohua Yan, Hanji Huang, Mingjun Zheng, Ke Ma, Xiaofei Cui, Dejie Lu, Li Zheng, Bo Zhu, Jianwen Cheng, Jinmin Zhao

**Affiliations:** 1 Guangxi Engineering Center in Biomedical Materials for Tissue and Organ Regeneration, The First Affiliated Hospital of Guangxi Medical University, Nanning, 530021 Guangxi China; 2 Guangxi Collaborative Innovation Center for Biomedicine, Guangxi Medical University, Nanning, 530021 China; 3 Department of Orthopaedics Trauma and Hand Surgery, The First Affiliated Hospital of Guangxi Medical University, Nanning, 530021 Guangxi China; 4 Department of Plastic & Cosmetic Surgery, The First Affiliated Hospital of Guangxi Medical University, Guangxi Medical University, Nanning, 530021 China; 5 International Joint Laboratory of Ministry of Education for Regeneration of Bone and Soft Tissues, The First Affiliated Hospital of Guangxi Medical University, Nanning, 530021 China; 6 Guangxi Key Laboratory of Regenerative Medicine, The First Affiliated Hospital of Guangxi Medical University, Nanning, 530021 China

**Keywords:** Human umbilical cord mesenchymal stem cells, Extracellular vesicles, Osteoarthritis, Macrophage, MicroRNA

## Abstract

Osteoarthritis (OA) is a degenerative illness that greatly impacts the life quality of patients. Currently, the therapeutic approaches for OA are very limited in clinical. The extracellular vesicles (EVs) derived from different mesenchymal stem cells displayed a prominent therapeutic effect on OA. But most EVs have limited resources and the risks of host rejection, immunological response, and etc. Human umbilical cord mesenchymal stem cells (hUCMSCs) hold the advantages of easy availability, minimal immune rejection, and excellent immunomodulatory effects, although hUCMSCs-EVs have seldom been applied in OA. Herein, we investigated the potential immunomodulatory and anti-inflammatory effects of hUCMSCs-EVs on the treatment of OA. In our results, the treatment of hUCMSCs-EVs promoted the polarization of M2-type macrophages and the expression of anti-inflammation-related cytokines (IL-10). Notably, the supernate of M2 macrophages induced by hUCMSCs-EVs inhibited the level of inflammation-associated factors in OA chondrocytes caused by IL-1β. Further, injection of hUCMSCs-EVs in the articular lumen ameliorated progression of OA and exerted chondroprotective potential based on the OA joint model created by the surgical transection of the anterior cruciate ligament (ACLT). In addition, we found five highly enriched miRNAs in hUCMSCs-EVs, including has-miR-122-5p, has-miR-148a-3p, has-miR-486-5p, has-miR-let-7a-5p, and has-miR-100-5p by High-throughput sequencing of miRNAs, with targeted genes mainly enriched in the PI3K-Akt signaling pathway. Furthermore, we also detected the protein abundance of hUCMSCs-EVs using liquidation chromatography with tandem quadrupole mass spectrometry (LC–MS/MS) analysis. Thus, our study indicates that hUCMSCs-EVs can alleviate cartilage degradation during the OA progression, mechanically may through delivering key proteins and modulating the PI3K-Akt signaling pathway mediated by miRNAs to promote polarization of M2 macrophage, exhibiting potent immunomodulatory potential. The current findings suggest that hUCMSCs-EVs might serve as a new reagent for the therapy of OA.

**Graphical Abstract:**

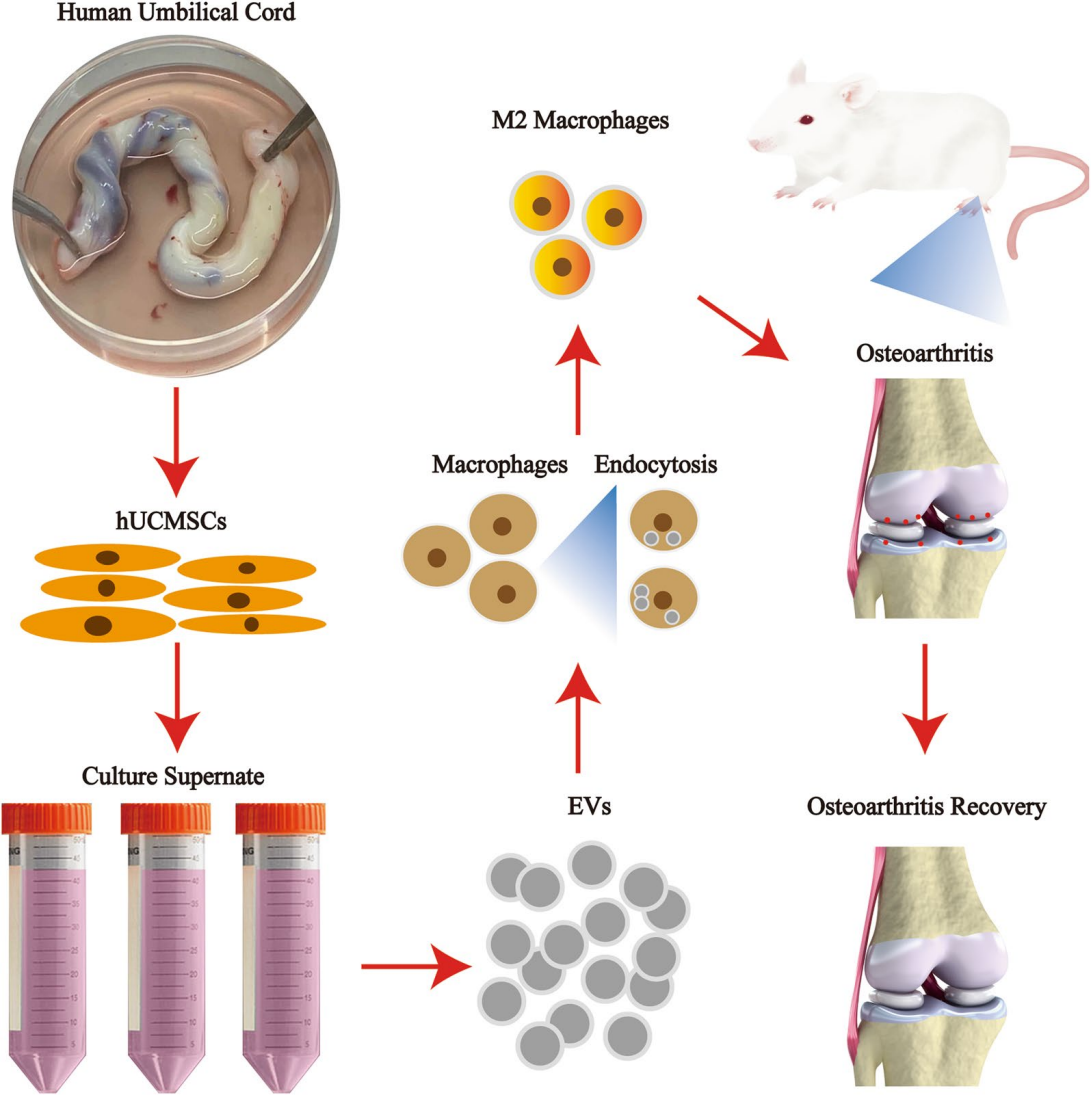

**Supplementary Information:**

The online version contains supplementary material available at 10.1186/s12951-021-01236-1.

## Introduction

Osteoarthritis (OA) is a chronic osteoarthropathy distinguished by chronic progressive degeneration of the articular cartilage and inflammation of the synovial joint [1]. It may elicit joint pains and dysfunctions, even severe disabilities for patients. Currently, the therapeutic approaches for OA are very limited in clinical, only medication and surgical treatment. However, pharmacological treatments only temporarily control inflammation and alleviate the pain but can not prevent cartilage destruction in OA. Surgical treatment such as arthroplasty or total joint replacement surgery also causes a great deal of stress on the patients with OA both physically and financially [2]. Although some materials or drug delivery systems for the treatment of OA being extensively studied and had been found to have a certain therapeutic effect, such as triamcinolone acetonide-encapsulated polymeric nanoparticles (TePNs) hydrogel system, ice-inspired lubricated drug delivery particles [3, 4], these materials were used to treat osteoarthritis mainly through loading chemical drugs, and long-term use of drugs will eventually produce certain side effects. Thus, it is necessary to develop new therapeutic strategies that can reduce inflammation and promote regeneration of the degenerated cartilage.

Recently, small extracellular vesicles (EVs) have emerged as an outstanding candidate for the application of cell-free therapy in degenerative disorders through immune regulation and tissue regeneration [5]. Small EVs are nano-sized membrane vesicles between 30 and 150 nm that can be secreted and released by multiple cell types and could affect the function of recipient cells through transferring bioactive components (lipid, microRNA, lncRNA, and specific proteins, etc.) between cells [6]. Increasing evidence has shown small EVs confer immunomodulatory and anti-inflammatory effects in various inflammatory disorders and tissue injury. For instance, cardiac progenitor cells-derived EVs maintain monocytes to enhance the repair and healing of injured hearts with anti-inflammatory/immunological regulation [7]. Macrophage-derived EVs ameliorate inflammatory pain regulation through USP5-mediated HDAC2/NRF2 axis [8]. Endothelial progenitor cells-derived EVs also have a potential therapeutic effect on drug-resistant glomerulonephritis as it preserved glomerular endothelial cells' integrity from complement- and cytokine-induced damage [9].

In recent years, researchers found that small EVs from mesenchymal stem cells (MSCs) displayed a prominent therapeutic effect on OA [10]. MSCs are a kind of cells with the potential of self-renewal and directed differentiation, which can differentiate into different cell types, such as osteoblast, adipocytes, and chondrocytes. Currently, MSCs have been broadly applied in cell-based treatments for clinical, preclinical, and tissue engineering applications due to their immunomodulation and regenerative properties [11]. It has been proved the paracrine factors of MSCs, mainly small EVs, contribute to the therapeutic potential of MSCs [12]. A series of studies confirmed that MSCs-derived EVs could effectively treat OA. Small EVs from human bone marrow-derived MSCs (hBMSCs-EVs) promoted cartilage repair by triggering extracellular matrix production in OA [13]. Small EVs originated from adipose-derived mesenchymal stem cells (ADMSCs-EVs) exerted chondroprotective roles in OA through suppressing inflammatory mediators and MMP activity and promoting anti-inflammatory cytokines [14]. In the study of the OA model induced by collagen, synovium MSCs-originated EVs or were also found to improve OA [15]. However, the resources of these MSCs are limited. Besides, most allogeneic adult MSCs have the risks of host rejection, immunological response, pro-tumorigenesis, and etc.

The EVs secreted by MSCs derived from the human umbilical cord (hUCMSCs-EVs) have become an excellent option for cell-free therapy due to hUCMSCs possess several intrinsic advantages. Compared to other tissue-derived MSCs, hUCMSCs are easily obtained from the human umbilical cord and can be isolated non-invasively at low cost [16]. Besides, it is a less ethical concern because the human umbilical cord is easy to access would usually be discarded tissue [17]. Most importantly, hUCMSCs seldom produce in vitro immune responses from allogeneic T cells. And they secrete a good deal of tolerance-related factors, including TGF-β1 and IL-10, indicating low immunogenicity and favorable immunomodulatory properties [18]. Previous studies have shown that hUCMSCs-EVs can be used to cure various illnesses associated with inflammation, such as collagen-induced arthritis, excessive inflammation induced by the severe burn, LPS-induced macrophage inflammation, inflammatory bowel disease, retinal inflammation [19–23]. But hUCMSCs-EVs have not been applied in OA therapy.

This study explored the therapeutic effects of hUCMSCs-EVs on OA based on the OA chondrocytes mediated by IL-1β in vitro and OA model induced by the surgical transection of the anterior cruciate ligament (ACLT) in vivo. We found that hUCMSCs-EVs alleviated the process of OA possibly by delivering critical proteins and miRNA like has-miR-122-5p, has-miR-148a-3p, has-miR-486-5p, has-miR-let-7a-5p, and has-miR-100-5p to activate the PI3K-Akt signaling pathway that promotes polarization of M2 macrophage, which is implicated in the modulation of inflammation and immunoreaction. This research may offer a valuable reference for the therapy of OA with hUCMSCs-EVs.

## Results

### Isolation and differentiation potential of hUCMSCs

**Fig.1:**
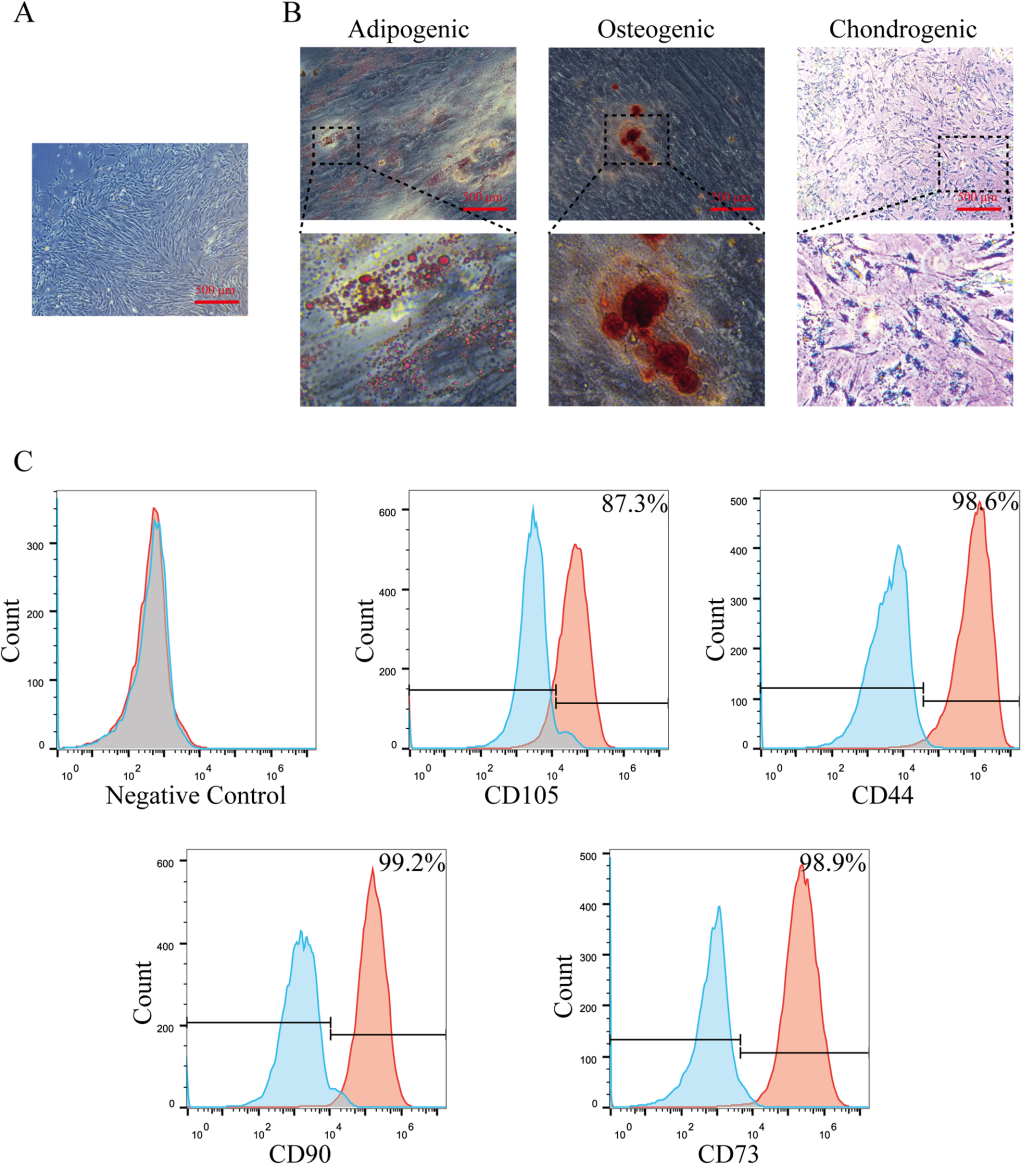
Characterization of human umbilical cord mesenchymal stem cells (hUCMSCs). **A** The hUCMSCs showed a long spindle morphology under the microscope; Scale bar: 500 μm. **B** The potential of hUCMSCs on differentiation of osteoblasts, adipocytes, and chondrocytes in the specified medium was identified by the Alizarin Red S staining, Oil Red O staining, and Alcian Blue staining, respectively; Scale bar: 500 μm. **C** Flow cytometry detection of the typical surface markers CD105, CD90, CD44, and CD73 in hUCMSCs; Blue curves: the isotype controls; red curves: the test samples

To obtain small extracellular vesicles from hUCMSCs, we first isolated and cultured MSCs from the human umbilical cord as mentioned in the “Methods and materials” section. The results of morphology showed that the hUCMSCs grew in a monolayer and exhibited fibroblast-like characteristics (spindle shape) (Fig. 1A). To further identify the hUCMSCs, we analyzed the differentiation potential of the extracted hUCMSCs by inducing hUCMSCs to differentiate into osteoblast, adipocytes, and chondrocytes. As shown in Fig. 1B, calcium phosphate deposition (dark orange spots) was observed in the cells cultured in complete osteogenesis differentiation medium by Alizarin Red S staining; lipid droplets (the red spots) were appeared in the cytoplasm of the cells cultured in complete adipogenesis differentiation medium by Oil Red O staining; production of proteoglycan (blue circle) was present in the cell cultured in differentiation medium for chondrogenesis by Alcian Blue staining. These results indicate that hUCMSCs are characterized by pluripotent differentiation. In addition, the markers related to mesenchymal stem cells were further confirmed by flow cytometry. The results showed that hUCMSCs were positive for CD105, CD44, CD90, and CD73 with 87.3%, 98.6%, 99.2% and 98.9%, respectively (Fig. 1C). These data suggest that the hUCMSCs were successfully extracted from the umbilical cord.

### Isolation and characterization of the extracellular vesicles derived from hUCMSCs

**Fig.2:**
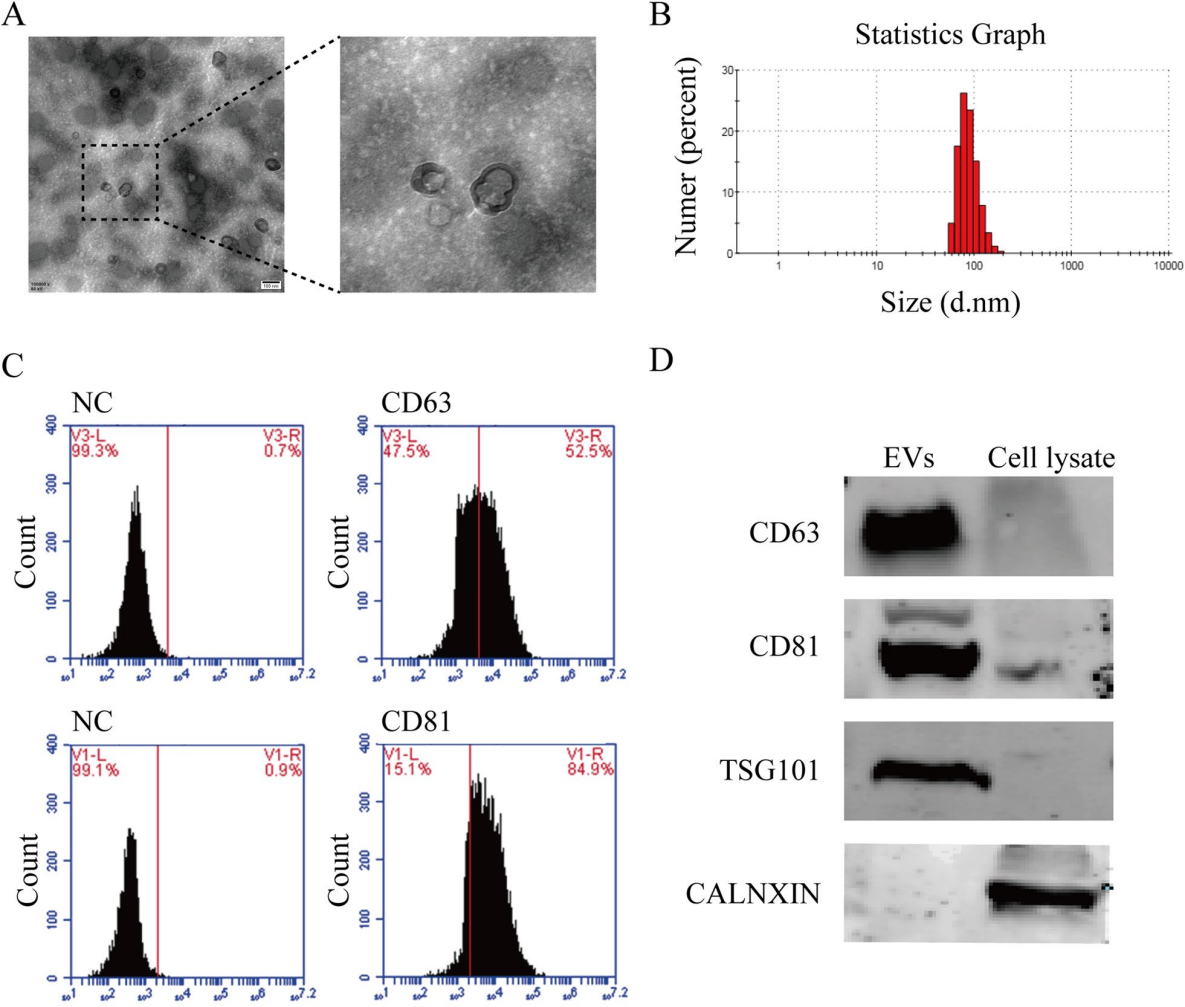
Determination of hUCMSCs-derived small extracellular vesicles (hUCMSCs-EVs). **A** Representative transmission electron microscopy image of hUCMSCs-EVs; Scale bar: 100 nm. **B** hUCMSCs-EVs size was assessed by Zetaszier Nano-ZS. **C** The typical surface markers CD63 and CD81 in hUCMSCs-EVs were detected by flow cytometric analysis. **D** hUCMSCs-EVs biomarkers, including CD63, CD81, TSG101, and hUCMSCs biomarkers CALNEXIN, were analyzed by western blot

Next, we isolated the EVs from the culture supernatants of hUCMSCs. As shown in Fig. 2A, the purified hUCMSCs-EVs were around a spherical shape with a hypodense center under the transmission electron microscopy. And the particle size of EVs also was verified to be 30 to 150 nm in diameter by Zetaszier Nano-ZS (Fig. 2B). Additionally, the biomarkers of the EVs also were detected in hUCMSCs-EVs by flow cytometry. The results of the flow cytometry assay displayed that hUCMSCs-EVs were positive for CD63 and CD81 with 52.5% and 84.9%, respectively (Fig. 2C). Moreover, we further detected the biomarkers of the EVs by western blot assay. As exhibited in Fig. 2D, the hUCMSCs-EVs positive expressed subsets of proteins that are commonly present in exosomes, such as CD63, CD81, and TSG101, but not expressed CALNXIN protein. In contrast, these protein expressions were not detected in the cell lysate, except for CALNXIN. Altogether, these results suggest that the small extracellular vesicles were successfully purified and identified from hUCMSCs.

### hUCMSCs-EVs effectively encourage the polarization of M2 macrophages in vitro and determination of optimal concentration for hUCMSCs-EVs

**Fig.3:**
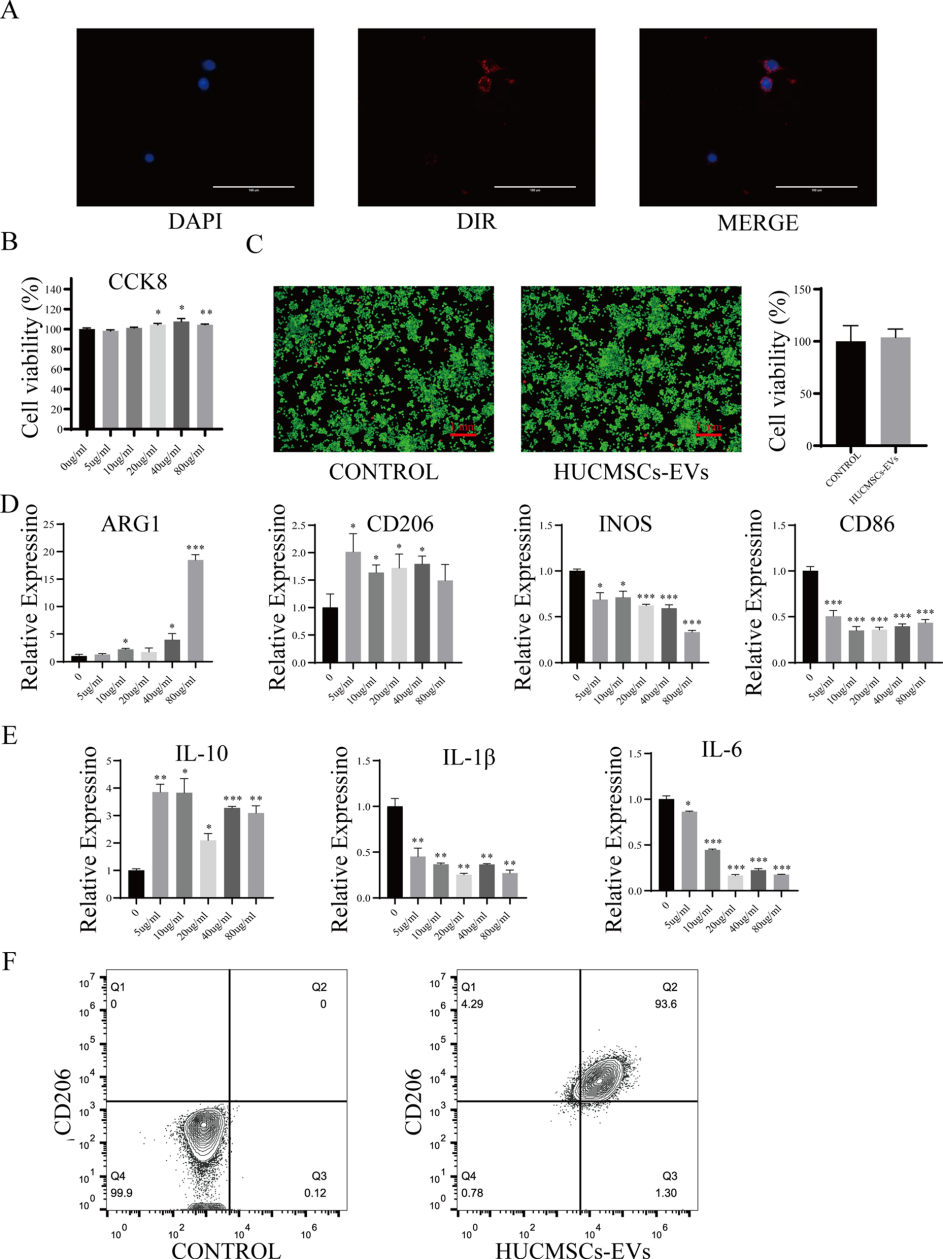
HUCMSCs-EVs effectively promote the polarization of M2 macrophages in vitro. **A** hUCMSCs-EVs were labeled with DIR, and uptake of DIR-labeled hUCMSCs-EVs by macrophages was observed by confocal microscope; Scale bar: 100 μm. **B** The proliferative effect of hUCMSCs-EVs on macrophages was measured by CCK-8 analysis. **C** The impact of hUCMSCs-EVs on the viability of macrophages was detected by the cell live/death experiment; green represents live cells while red represents dead cells; Scale bar: 1 mm. **D** Relative mRNA expression of the critical genes ARG1, CD206, INOS, and CD86 was determined in polarized macrophages by quantitative RT-PCR analysis; the data of triplicate experiments are presented as mean ± S.D. *p < 0.05, **p < 0.01, ***p < 0.001. **E** Relative mRNA expression of the related cytokines IL-10, IL-1, and IL-6 were determined in polarized macrophages by quantitative RT-PCR analysis; the experiment was conducted triplicate; *p < 0.05, **p < 0.01, ***p < 0.001. **F** The typical surface markers in M2-polarized macrophages induced by hUCMSCs-EVs were detected by Flow cytometry analysis

The polarized phenotype of macrophage has been proved to be associated with the progression of OA [24]. Therefore, we first investigated the influence of hUCMSCs-EVs on the polarization of macrophages. To test whether hUCMSCs-EVs could act on macrophages, we first examined the cellular uptake of hUCMSCs-EVs through co-culturing macrophages with DiR-labeled hUCMSCs-EVs for 24 h. As expected, we observed that DiR-labeled hUCMSCs-EVs were utterly swallowed in the macrophages by confocal microscopy (Fig. 3A). To determine the optimal concentration of hUCMSCs-EVs for M2 macrophages polarization, we added the different doses of hUCMSCs-EVs (0, 5, 10, 20, 40, and 80 μg/mL) into the cultured macrophages. Firstly, we performed a CCK-8 experiment to test the influence of hUCMSCs-EVs on the proliferation ability of macrophages. As shown in Fig. 3B, the cell proliferation was not significantly changed in hUCMSCs-EVs incubation for 24 h at the concentration range from 0 to 10 μg/mL. Still, the proliferation ability of cells was slightly improved at 80 μg/mL concentrations, which indicated hUCMSCs-EVs did not show apparent cytotoxicity. In addition, the cell live/death experiment also further confirmed the results, in which, significant change in cell death was not observed under the 80 μg/mL of hUCMSCs-EVs treatment (Fig. 3C). Next, we detected the influence of these different doses of hUCMSCs-EVs on M2 macrophages polarization. As shown in Fig. 3D, hUCMSCs-EVs stimulation upregulated mRNA expression of Arg1 and CD206, which are M2 macrophages markers, while downregulated M1 macrophages markers (INOS and CD86) remarkably, indicating the hUCMSCs-EVs effectively promote the polarization of macrophages toward M2 phenotype macrophages rather than M1 phenotype. Moreover, to evaluate the anti-inflammatory functions of hUCMSCs-EVs via macrophage repolarization, we also examined the cytokines produced by M1 and M2 macrophages. The data of RT-PCR analysis exhibited that the expression level of anti-inflammation-related factor, IL-10, was increased. In contrast, the expression level of pro-inflammation-related factors, IL-1 and IL-6, was decreased dramatically after hUCMSCs-EVs administration (Fig. 3E). These data suggest that stimulation of hUCMSCs-EVs inhibits and facilitates the production of pro-inflammation- and anti-inflammation-related factors in macrophages, respectively. Notably, among these concentrations of hUCMSCs-EVs, the highest polarization of M2 macrophages was observed at 80 μg/mL of concentration, and cells treated with 80 μg/mL of hUCMSCs-EVs secreted the highest level of IL-10 and the lowest level of IL-1 and IL-6. The flow cytometry results also further confirmed that 80 μg/mL of hUCMSCs-EVs effectively foster the polarization of M2 macrophages, as indicated by the higher proportion of CD206^+^ and F4-80^+^ macrophages (72.8%) appeared in the hUCMSCs-EVs-treated group compared with the control (Fig. 3F). Thus, 80 μg/mL of hUCMSCs-EVs was chosen as the optimal concentration employed in the following experiments. Notably, we also detected the efficiency of hUCMSCs-EVs at 80 μg/mL on human macrophages, THP-1 using RT-PCR analysis. The result showed that M1 macrophages markers (TNF-α, IL-1β, and IL-6) were significantly down-regulated while M2 macrophages markers (CD206, CD163, and IL-10) were dramatically down-regulated in THP-1 cells after stimulation of hUCMSCs-EVs (Additional file 1: Fig. S1). These data indicated that hUCMSCs-EVs can significantly drive the polarization of macrophages to M2 instead of M1 polarization.

### Protective effect of M2 macrophages induced by hUCMSCs-EVs on OA chondrocytes mediated by IL-1β in vitro

**Fig.4:**
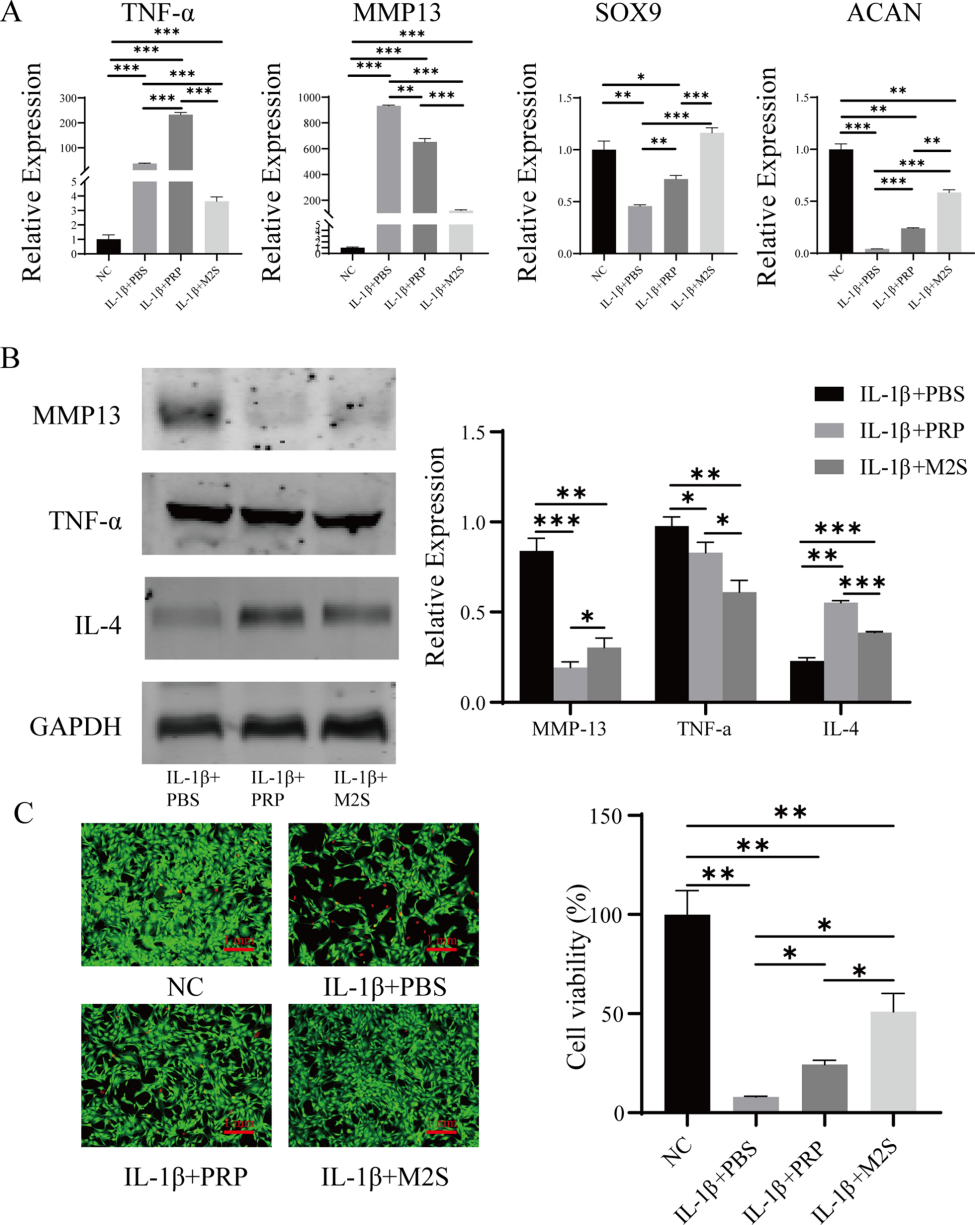
Protective effect of M2 macrophages induced by hUCMSCs-EVs on OA chondrocytes in vitro. **A** IL-1β-induced OA chondrocytes were co-cultured with the supernatant of M2 macrophages (M2S) induced by hUCMSCs-EVs, or platelet-rich plasma (PRP) for 48 h, relative mRNA expression of the key genes TNF-α, MMP13, SOX9, and ACAN was measured by quantitative RT-PCR analysis; the experiment was performed triplicate; *p < 0.05, **p < 0.01, ***p < 0.001. **B** Western blot was performed to evaluate the expression of TNF-α, MMP13, and IL-4 proteins in PBS, M2S, or PRP-treated OA chondrocytes; GAPDH was employed as the loading control; *p < 0.05, **p < 0.01, ***p < 0.001. **C** The influence of M2S or PRP on the viability of chondrocytes was detected by the cell live/death experiment; green represents live cells while red represents dead cells; Scale bar: 1 mm

To investigate the protective effects of polarized M2 macrophages induced by hUCMSCs-EVs on OA chondrocytes, we first investigated the biosafety of hUCMSCs-EVs on chondrocytes in vitro, The CCK8 and the cell live/death experiment showed that the viability of chondrocytes did not change significantly after treatment with hUCMSCs-EVs (Additional file 2: Fig. S2), indicating that hUCMSCs-EVs have good biosafety on chondrocytes. Next, we established a model of OA-like chondrocytes by co-incubation of chondrocytes with 10 ng of IL-1β. After 48 h of IL-1β induction, OA chondrocytes were co-cultured with the supernatant of M2 macrophages (M2S) caused by hUCMSCs-EVs, and platelet-rich plasma (PRP) was employed as a positive control [25, 26]. As exhibited in Fig. 4A, M2S treatment significantly increased expression of chondrocyte-specific marker genes Acan, Sox9. Still, the upregulated catabolic gene MMP-13 and inflammatory marker gene TNF-α caused by IL-1β were inhibited dramatically after treatment of M2S. Furthermore, the expression of MMP-13 and TNF-α proteins in the M2S treatment group was considerably lower than the PBS treatment group by western blot assay. At the same time, the level of anti-inflammation-related cytokine IL-4 was significantly elevated after M2S treatment (Fig. 4B). Notably, we also observed the treatment of hUCMSCs-EVs can inhibit apoptosis of IL-1β-induced OA chondrocytes by live/death assay (Fig. 4C). These results reveal that paracrine secretion of polarized M2 macrophages induced by hUCMSCs-EVs has a significant protective effect on OA chondrocytes in vitro.

### HUCMSCs-EVs could lessen the progression of ACLT-induced OA in vivo

**Fig.5:**
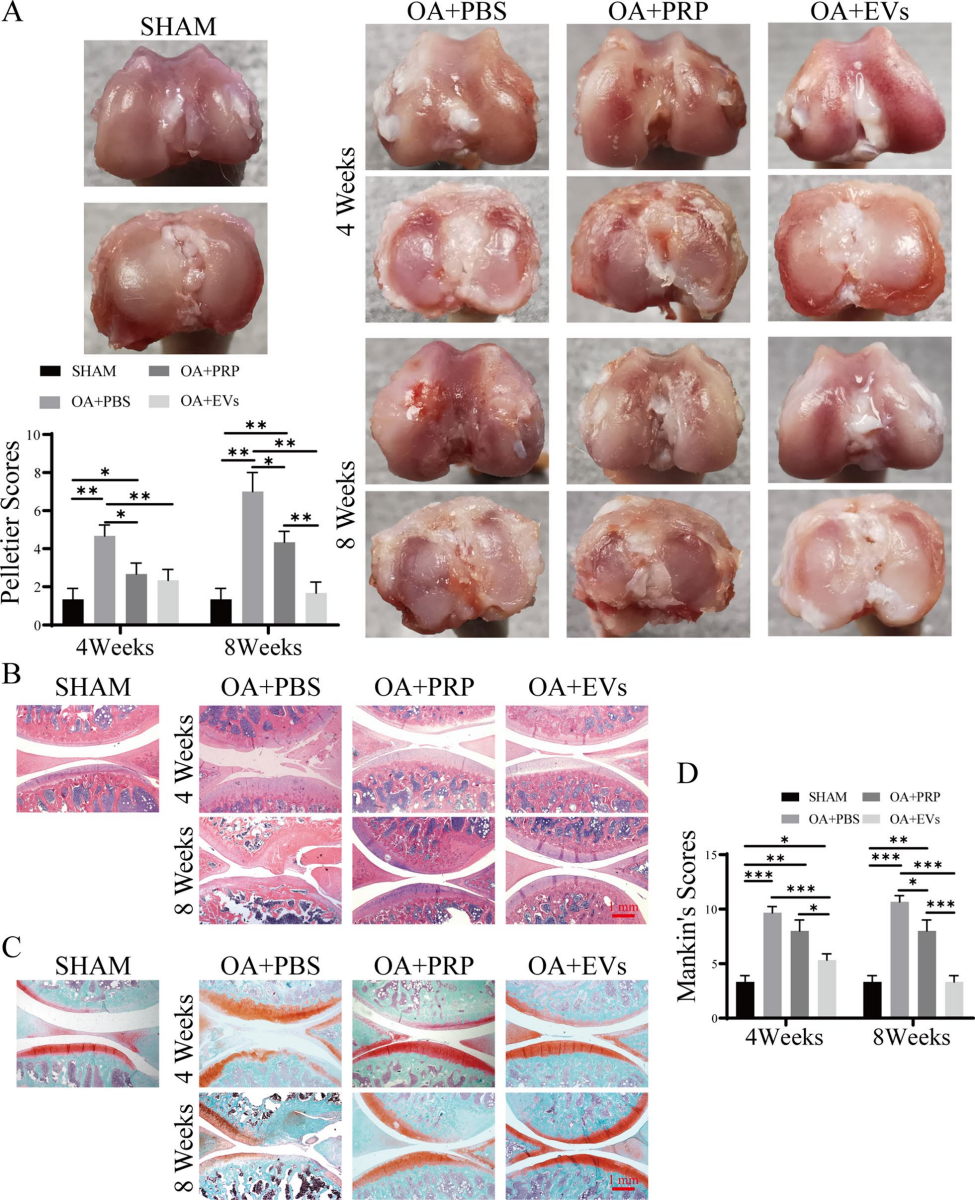
Therapeutic efficacy of hUCMSCs-EVs in OA rat model. **A** Macroscopic observation of articular cartilage for OA with PBS, hUCMSCs-EVs or PRP treatment, and the corresponding macroscopic scores; *p < 0.05, **p < 0.01, ***p < 0.001. H&E staining (**B**), Safranin-O Fast green staining (**C**), Histological score (**D**) on effects of hUCMSC-Evs for OA treatment; Scale bar: 1 mm; *p < 0.05, **p < 0.01, ***p < 0.001

To further investigate the therapeutic functions of hUCMSCs-EVs on OA in vivo, we generated the OA rat model by ACLT, a well-accepted progressive OA model in vivo. The gross macroscopic changes in articular cartilage were observed at 4 weeks and 8 weeks after operations. As shown in Fig. 5A, compared to the normal group that the tibial plateau and the femoral condyle possess the integrated and smooth cartilage surface, the cartilage surface of the model group exhibited apparent erosion and discoloration of yellowish, even part of the subchondral bone falls off, indicating the typical morphological changes of OA cartilage in rats. In the hUCMSCs-EVs-treated group, the OA cartilage was significantly lessened and showed standard cartilage color; even there were still small fissures into superficial layers. However, in the group of treatment with PBS, there was no noticeable improvement for the surfaces of articular cartilage of OA rats. Furthermore, the results of H&E staining also showed that the PBS-treated rats still displayed severe erosion in the calcified cartilage. Still, the cartilage surface became smooth and intact in the hUCMSCs-EVs and PRP treatment group. And more notably, the hUCMSCs-EVs-treated group exhibited more smooth and intact cartilage surface, similar to those of normal knee cartilage compared with the PRP-treated group, especially in 8 weeks (Fig. 5B). Extensive proteoglycan loss was observed in the PBS treatment group using Safranin O fast green staining (Fig. 5C). However, treatment of hUCMSCs-EVs and PRP increased proteoglycan compared with PBS groups in 4 and 8 weeks. Interestingly, among hUCMSCs-EVs and PRP groups, the production of proteoglycan in the hUCMSCs-EVs group was greater than the PRP group, indicating hUCMSCs-EVs could ameliorate the OA progression caused by ACLT, and the effect of repair is more robust than PRP. Furthermore, the OARSI grade further revealed that the hUCMSCs-EVs-treated group showed the lowest scores in all the groups (Fig. 5D). These data indicate that hUCMSCs-EVs could strongly inhibit the process of OA in vivo.

**Fig.6:**
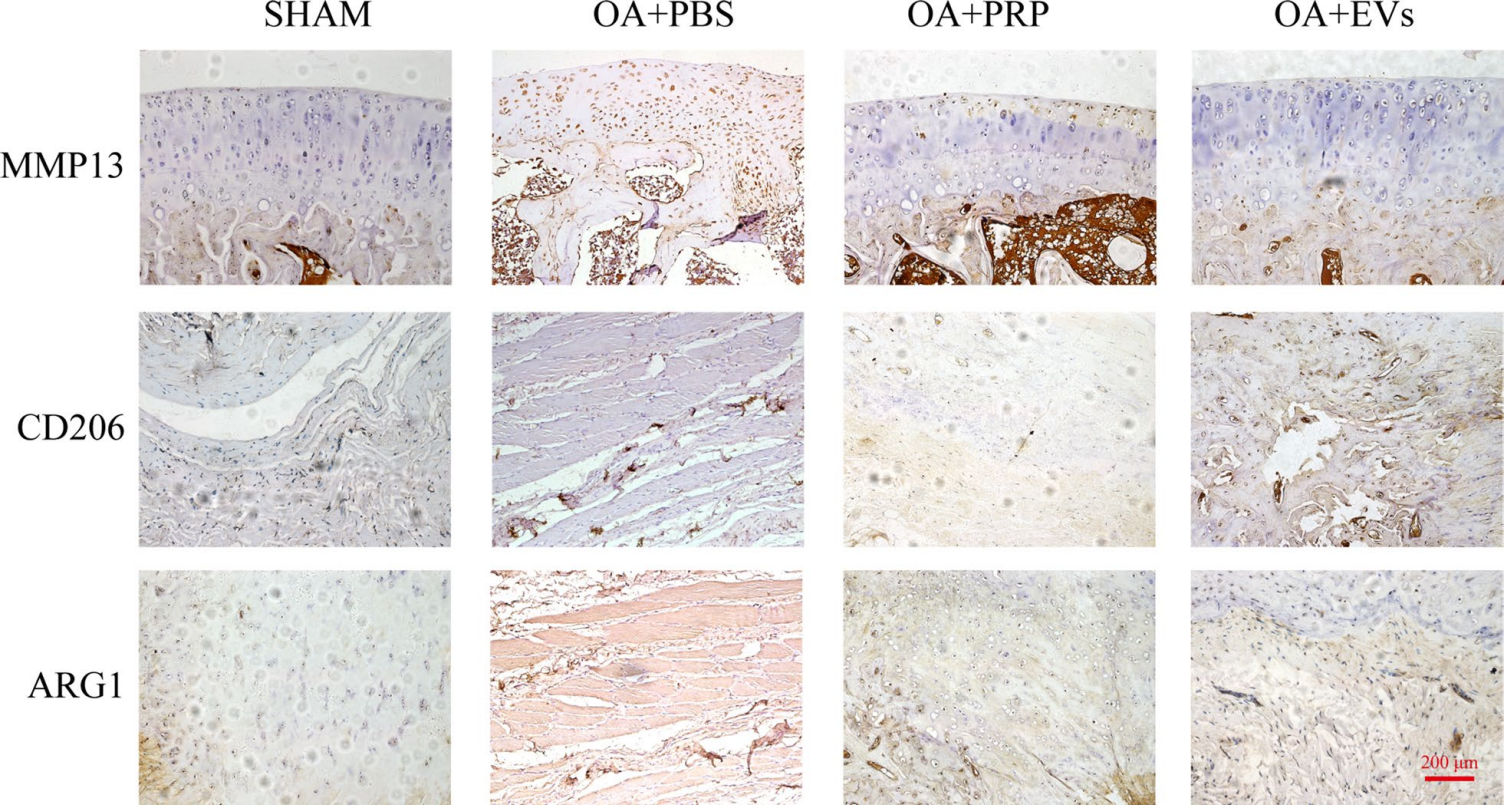
Immunohistochemical staining was performed to detect expression of ARG1, CD206, and MMP13 in articular cartilage of OA rat model after treatment with indicated reagents; Scale bar: 200 μm

Given M2 macrophages have anti-inflammatory functions that contribute to tissue repair and inflammation resolution [27]. We further analyze the immunomodulation ability of hUCMSCs-EVs on M2 macrophage infiltration in the ACLT-induced OA model by immunostaining. As shown in Fig. 6, the injection of hUCMSCs-EVs strongly promoted the level of M2 macrophage infiltration in the ACLT-induced OA model compared to the control group, as evidenced by increased expression of ARG1 and CD206 appeared in the synovial tissue of the hUCMSCs-EVs treatment group. Meanwhile, we also detected the expression of MMP13 in the ACLT-induced OA model. The results of immunostaining showed that surgery induces the upregulation of MMP13 in articular cartilage; however, administration of hUCMSCs-EVs or PRP effectively diminished the percentage of MMP13-positive chondrocytes in ACLT-induced OA rats compared to treatment with PBS. Notably, compared with PRP, hUCMSCS-EV also significantly reduced the rate of MMP13 positive chondrocytes (Fig. 6). These findings suggest that hUCMSCs-EVs treatment effectively inhibits the inflammation and protects against the degradation of cartilage in the ACLT-induced OA model partially through inducing polarization of M2 macrophage.

### hUCMSCs-EVs miRNA profiling and their putative functions by bioinformatics analysis

**Fig.7:**
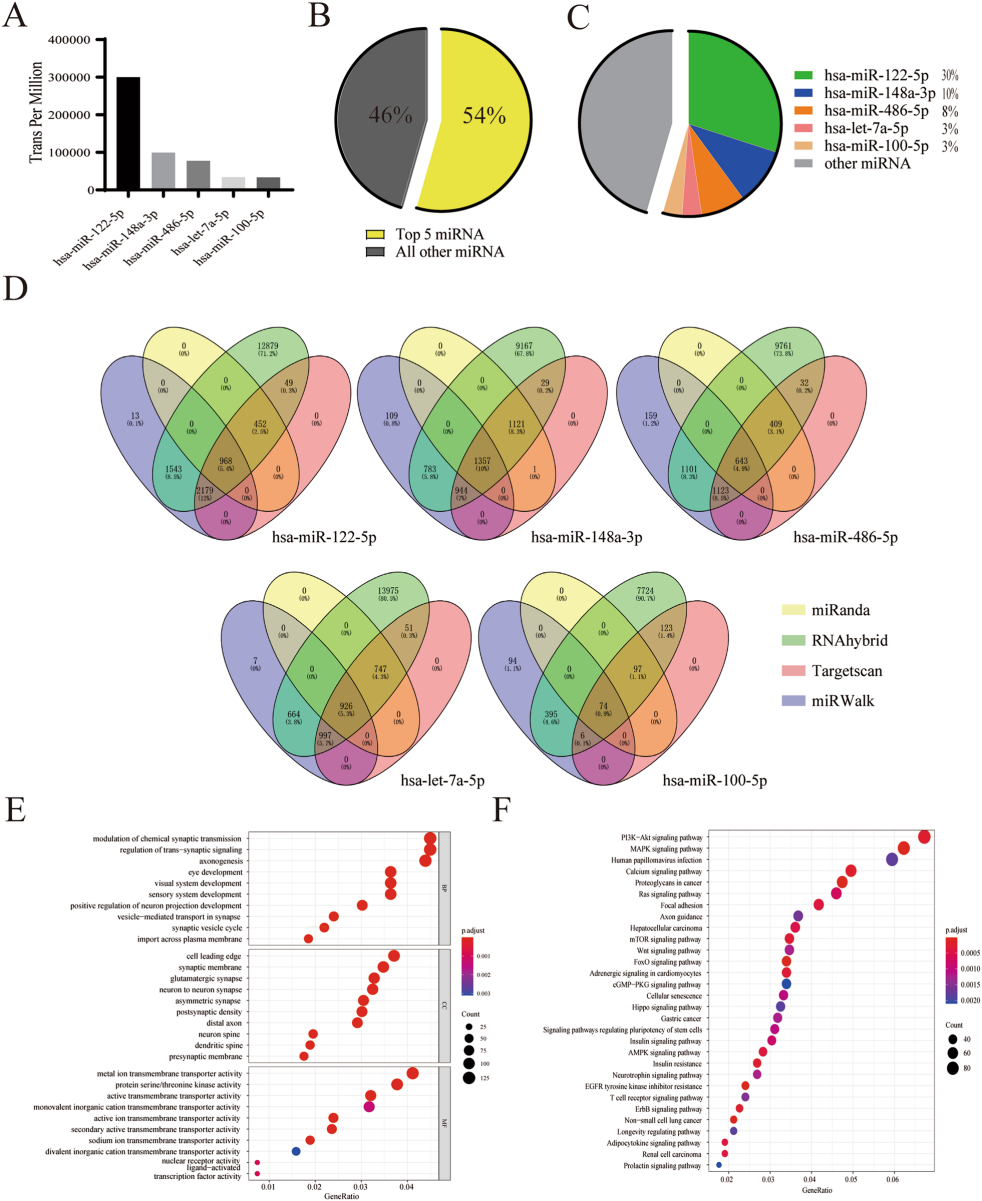
HUCMSCs-EVs miRNA profiling and their putative functions by bioinformatics analysis. **A** The five most abundant hUCMSCs-EVs miRNAs were exhibited by the histogram. **B** The proportion of the five most abundant hUCMSCs-EVs miRNAs in total miRNAs. **C** The ratio of each of the five most abundant hUCMSCs-EVs miRNAs in total miRNAs. **D** Venn diagram for the intersection of target genes predicted by the five most abundant hUCMSCs-EVs miRNAs. **E** The top 30 most enriched Gene Ontology (GO) terms for these target genes by GO analysis. **F** The top 30 most enriched pathways for these target genes by KEGG pathway analysis

As EVs exert their biological effects mainly through the delivery of bioactive molecules, such as miRNAs, to explore potential molecular mechanisms of the hUCMSCs-EVs for the treatment of OA, we profiled and quantified the miRNA expression of hUCMSCs-EVs through high-throughput miRNA-sequencing. According to the sequencing results, the top 5 known miRNAs (has-miR-122-5p, has-miR-486-5p, has-miR-148a-3p, has-miR-let-7a-5p, and has-miR-100-5p) identified in hUCMSCs-EVs were ordered based on the transcripts per million (TPM) (Fig. 7A). The five most abundant miRNAs accounted for 54% of the total miRNA tags (Fig. 7B); among these five miRNAs, has-miR-122-5p, has-miR-148a-3p, has-miR-486-5p, has-miR-let-7a-5p, and has-miR-100-5p accounted for 30%, 10%, 8%, 3% and 3% of the total miRNAs, respectively (Fig. 7C). The sequencing data suggested that hUCMSCs-EVs displayed a distinctive and ordered style, in which specific miRNA cargos were selectively packaged from hUCMSCs. To further investigate the function of hUCMSCs-EVs miRNAs, we carried out functional enrichment analyses for these five most abundant miRNAs. The target genes of the acquired five most abundant miRNAs were predicted by miRanda, RNAhybrid, miRWalk, and Targetscan. And the target genes of each miRNA were obtained by the intersection of prediction results of four databases. After collecting target genes of all five miRNAs, a total of 3968 genes were acquired as the result of the target gene prediction (Fig. 7D). Next, the functions of these predicted target genes were investigated by performing Gene Ontology (GO) and KEGG pathway analyses. Based on the GO database, these predicted target genes were primarily enriched in the regulation of neuron projection development, neuronal cell body, and protein serine/threonine kinase activity in the biological process (BP), molecular function (MF), and cellular component (CC), respectively (Fig. 7E). The KEGG pathway investigation indicated that these target genes were mainly enriched in the PI3K-Akt signaling pathway, Human papillomavirus infection, MAPK signaling pathway, Calcium signaling pathway, and Proteoglycans in cancer. In particular, the PI3K-Akt signaling pathway exhibited the most obvious advantage, which enriched the most target genes (Fig. 7F), indicating that the PI3K-Akt signaling pathway may play an essential role in the therapeutic effects of hUCMSCs-EVs on OA. Collectively, these data suggest that hUCMSCs-EVs attenuated the development of OA may through transferring miRNA into recipient cells, thereby modulating the PI3K-Akt signaling pathway.

### hUCMSCs-EVs protein profiling and their putative functions by bioinformatics analysis

**Fig. 8:**
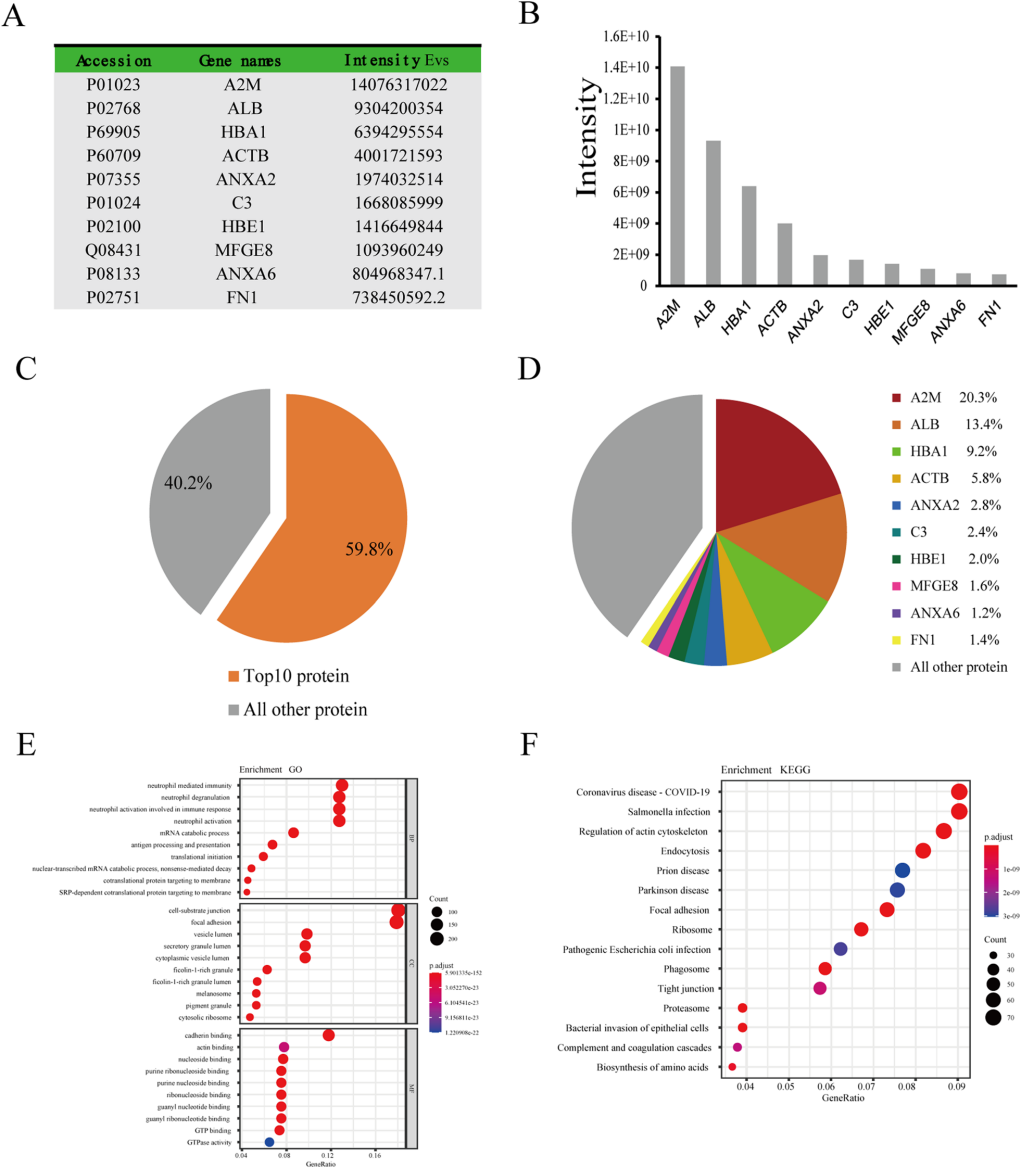
HUCMSCs-EVs protein abundance and their putative functions. **A** The ten most abundant hUCMSCs-EVs proteins determined by LC–MS/MS Analysis. **B** The ten most abundant hUCMSCs-EVs proteins were arranged and exhibited by the histogram. **C** The proportion of the ten most abundant hUCMSCs-EVs proteins in total miRNAs. **D** The ratio of each of the ten most abundant hUCMSCs-EVs proteins in total miRNAs. **E** The top 30 most enriched GO terms for these proteins by GO analysis. **F** The top 15 most enriched pathways for these proteins by KEGG pathway analysis

Since proteins are also the primary component in EVs, we profiled the protein abundance of hUCMSCs-EVs using liquidation chromatography with tandem quadrupole mass spectrometry (LC–MS/MS) analysis. According to the results of protein abundance analysis, we quantified a total of 1237 proteins in the hUCMSCs-EVs. The ten most abundant proteins were A2M, ALB, HBA1, ACTB, ANXA2, C3, HBE1, MFGE8, ANXA6, and FN1, which were arranged according to the intensity and displayed on a histogram (Fig. 8A, B). The top ten proteins accounted for 59.8% of the total abundance of proteins (Fig. 8C). Among these ten proteins, A2M protein accounted for 20.3% of the total quantity of proteins, followed by ALB protein (13.4%) and HBA1 protein (9.2%) (Fig. 8D). Next, we performed GO and KEGG enrichment analyses to gain further insight into the functional roles of the quantified proteins from hUCMSCs-EVs. According to the results of GO analysis, these identified proteins deriving from hUCMSCs-EVs were mainly enriched in the following items, neutrophil mediated immunity, neutrophil degranulation, and neutrophil activation involved in immune response in the category of BP. In the class of MF, the three items with the most abundant protein were cell-substrate junction, focal adhesion, and vesicle lumen. In the category of CC, cadherin binding, actin binding, and nucleoside binding were the three most significant items (Fig. 8E). Furthermore, the results of KEGG enrichment revealed that these proteins were principally enriched in the following pathways, including Coronavirus disease-COVID-19, Salmonella infection, Regulation of actin cytoskeleton, Endocytosis, and Prion disease (Fig. 8F). These data suggest that these crucial proteins may cooperate with miRNA-regulated signaling pathways to participate in the mechanism of hUCMSCs-EVs in the treatment of OA.

## Discussion

In this research, we demonstrated that hUCMSCs-EVs could effectively weaken the OA development in the ACLT-induced OA models mechanically through delivering miRNAs and key proteins to administrate the PI3K-Akt signaling pathway that promotes the polarization of M2 macrophages, thereby reducing the inflammatory response.

Recently, more and more evidence showed that EVs from different cell sources play an increasingly prominent role in the field of OA treatment. And hUCMSCs-EVs have been shown to have positively therapeutic effects and frequently be applied in regenerative medicine and various diseases treatment, such as Kidney diseases, Alzheimer's disease, Nerve injury–induced pain and Inflammatory bowel disease [21, 28]. These investigations suggested that potential clinical applications of hUCMSCs-EVs in various diseases treatment. However, the influence of hUCMSCs-EVs on the progression and treatment of OA remains unknown. Here, we researched the therapeutic effect of hUCMSCs-EVs in the ACLT-induced OA rat models. Our data showed that intra-articular injection of hUCMSCs-EVs effectually attenuates the process of OA. And the therapeutic roles of hUCMSCs-EVs on OA were superior to those of PRP, which is applied with increasing frequency to treat musculoskeletal pathologies, such as OA, by intra-articular injection [29]. These results are consistent with those of EVs derived from BMMSCs, ADSCs, and ESCs on the treatment of OA [30–32]. Therefore, together with our results, these findings further prove that the EVs derived from different stem cells appear to possess a special effect of alleviating OA.

It is well documented that inflammation is regarded as a prominent feature of OA pathogenesis. The progress of OA is accompanied by chondrocyte apoptosis and matrix degradation that is mainly due to secretion of the inflammation-related factors IL-6, TNF-α, and IL-1β, and rapid up-regulation of the catabolic factors such as MMP13 [33]. While, infiltration of macrophages was demonstrated to be the primary source of raised levels of inflammation-related factors (TNF-α, IL-6, IL-1β) in OA mouse models [34]. Thus, targeting macrophage polarization could be an effective strategy for regulating the process of inflammation to prevent and diminish the development of OA. Recently, the anti-inflammation effect of hUCMSCs-EVs via M2 polarization has been reported in several diseases. For example, Sun [35] et al. showed that hUCMSCs-EVs foster the transformation of macrophages from M1 phenotype to M2 phenotype, thereby weakening inflammation at the injury site and accelerating the rehabilitation of spinal cord injury. The hUCMSCs-EVs attenuate inflammatory responses and the alveolar injuries in diffuse alveolar hemorrhage (DAH) mice by activating M2 macrophage [36]. However, the anti-inflammation effect of hUCMSCs-EVs via M2 polarization in OA chondrocytes remains to be clarified. In the present study, we examined the impact of hUCMSCs-EVs on macrophage polarization and secretion of polarized M2 macrophages in IL-1β-induced OA chondrocytes in vitro. Interestingly, we found that hUCMSCs-EVs can promote the conversion of macrophage towards the M2 phenotype, decrease the secretion of the pro-inflammation-related factors TNF-α, IL-1, and IL-6, and augment the production of the anti-inflammatory cytokine IL-10. In vivo, hUCMSCs-EVs treatment markedly promoted the proportion of M2 macrophages and diminished the infiltration of inflammatory cells, as demonstrated by ARG1 and CD206-positive cells were significantly increased in synovial tissues. These data was consistent with previous findings showing that BMSCs-derived EVs can facilitate the transformation of RAW264.7 from M1 phenotype to M2 phenotype, down-regulate the level of pro-inflammatory factors TNF-α, IL-1β, and IL-6, and up-regulate the level of the anti-inflammatory cytokine IL-10 [37]. The recent data also showed that rat adipose-originated stem cells-derived EVs can facilitate polarization of M2 macrophage to ameliorate cardiac damage after myocardial infarction [38]. Human adipose-originated stem cells-derived EVs can prevent cartilage degeneration and reduce OA progression by inhibiting the infiltration of M1 macrophages into the synovium [39]. In addition, we also found that supernatant of M2-polarized macrophages induced by hUCMSCs-EVs effectively elevated the expression of Sox9 and ACAN, inhibited the IL-1β-mediated expression of MMP13, and secretion of cytokine TNF-α in chondrocytes. These findings agree with the function of BMSCs-derived EVs revealed by Zhang et al. [37]. The co-culture of M2 type macrophages induced by BMSCs-EVs with chondrocytes revealed that M2 type macrophages augmented the expression of collagen II and Sox9, while reduced expression of collagen X and Runx2. Xie et al. [40], also showed that co-culture of OA chondrocytes with polarized M2 macrophages diminished expression of OA-associated proteases (MMP3, MMP13, and ADAMTS5) but increased expression of chondrocyte-specific genes (COL2A1 and ACAN). Thus, our results provide evidence of hUCMSC-EVs ability to reduce inflammation response in OA might through the M2 macrophage-dependence mechanism.

Recently, different nucleic acids have been found in EVs, including mRNAs, non-coding RNAs, and miRNAs. Especially miRNAs, which are demonstrated as the key component of EVs, play critical roles in OA progression through modulating inflammatory response, chondrocyte survival, and extracellular matrix deposition [41]. These miRNAs carried by EVs can be taken up by recipient cells and subsequently exert important biological function in recipient cells. Therefore, it is important to clarify the miRNAs content of hUCMSCs-EVs to use hUCMSCs as a therapeutic strategy in OA. In this study, we conducted a high-throughput miRNA-sequencing to profile and quantify the miRNA expression of hUCMSCs-EVs. We found that five miRNAs with the most abundant contents in hUCMSCs-EVs are has-miR-122-5p, has-miR-486-5p, has-miR-148a-3p, has-miR-let-7a-5p, and has-miR-100-5p. We further revealed the target genes of these five miRNAs are primarily enriched in the PI3K-Akt signaling pathway through bioinformatic analysis. Recently, the PI3K-Akt signaling pathway has been revealed to have an essential role during the development of OA [42]. Interestingly, PI3K and Akt activation has been demonstrated to be a critical step toward activation of M2 macrophages [43], indicating that the PI3K-Akt signaling pathway is a central node for administrating the polarization of macrophages. Thus, we speculated that hUCMSCs-EVs attenuated the process of OA probably via transferring key miRNA into macrophage to modulate the PI3K-Akt signaling pathway, thereby initiating the transcription of some M2 macrophage-related functional genes and finally promoting M2 polarization. Except for miRNAs, we also detected protein component of hUCMSCs-EVs and found that the ten most abundant proteins in hUCMSCs-EVs were A2M, ALB, HBA1, ACTB, ANXA2, C3, HBE1, MFGE8, ANXA6, and FN1. Recent studies have revealed MFGE8 can alleviate inflammation by driving M2 polarization [44–46]. In addition, MFGE8 has been found to significantly enhance the expression of PI3K-Akt pathway-related components in the study of traumatic brain injury [47]. So we speculate that these key proteins likely join the regulation mechanism of hUCMSC-EV in OA therapy together with the PI3K-Akt signaling pathway. Although our study demonstrates that hUCMSCs-EVs effectively prevent OA progression and further illuminates hUCMSCs-EVs enriched miRNAs and proteins, the precise mechanism of their effects on OA therapy is not fully understood, especially the specific function of each miRNA and protein. Therefore, further detailed explorations are required to determine the key internal contents of hUCMSCs-EVs and specific molecular mechanisms on OA.

In conclusion, our study demonstrated that hUCMSCs-EVs could effectively alleviate OA progression likely via transferring key proteins and miRNAs to regulate the PI3K-Akt signaling pathway, thereby promoting the polarization of M2 macrophages phenotype, which modulates inflammatory and immune reactivity. Our current findings shed light on further approaches to investigate the clinical usage of hUCMSCs-EVs for the treatment of OA.

## Methods and materials

### Preparation and culture of hUCMSCs

All procedures involving human umbilical cords in this research were approved by the Medical Ethic Committee of The First Affiliated Hospital of Guangxi Medical University. And the informed consents were finished by all participants involved in the research before obtaining the umbilical cords. The fresh human umbilical cords were acquired under sterile conditions from healthy donors following full-term cesarean operation in The First Affiliated Hospital of Guangxi Medical University. Extraction of the MSCs from the human umbilical cords as described previously [48]. Briefly, the blood vessels in the umbilical cords are first removed and subsequently minced to obtain Wharton's jelly. Fragments that were cut into pieces one mm^3^ in size were plated to a petri dish, and Dulbecco's modified Eagle's medium (DMEM)/Basic (Hyclone) with 10% fetal bovine serum (FBS, Gemini) were added into the dish and incubated in an incubator with 5% CO_2_ at 37 °C. Adherent cells were sub-cultured when they reached 90% confluence in the culture dish, and the culture medium was refreshed twice 1 week. The hUCMSCs from passages 3 to 5 were applied in this study. To assess the ability of hUCMSCs on differentiation of adipocytes, osteocytes, and chondrocytes, the hUCMSCs were grown with adipogenic-, osteogenic-, or chondrogenic-inducing medium for 14 days. And then, the alizarin red staining, alcian blue staining, and oil red O staining were conducted to confirm osteocytes, chondrocytes, and adipocytes differentiation, respectively.

### Extraction and characterization of extracellular vesicles derived from hUCMSCs

For extraction of extracellular vesicles (EVs), the hUCMSCs (passage 3) were incubated with an FBS-free medium for 48 h; subsequently, the culture supernatants were gathered. The gradient centrifugation was applied to isolate the extracellular vesicles. In brief, the centrifugal force of 1000×*g*, 10,000×*g*, and 120,000×*g* for 20 min, 30 min, and 90 min, respectively, at 4 °C was set at the ultracentrifuge (Beckman Coulter Optima L-80 XP) to centrifuge the culture supernatants. 100 μL of PBS was applied to resuspend the final extracellular vesicles pellet and stored at − 80 °C. The morphology of hUCMSCs-EVs was confirmed using transmission electron microscopy (TEM; HITACHI). Subsequently, a bicinchoninic acid protein quantification kit (Thermo Scientific, 23225) was employed to test the extracellular vesicles concentration. A light-scattering technique, nanoparticle tracking analysis (NTA; ZetaView PMX 110), was used to test the particle distribution and zeta potential. Surface characteristic proteins CD63, TSG101, and CD81 were further examined through western blot assay.

### Culture and polarization of macrophages by stimulation with hUCMSCs-EVs

The macrophages were plated in 10 cm dish containing the corresponding complete medium and grown in an incubator, at which the level of CO_2_ was set to 5% and the temperature to 37 °C. To test the effects of hUCMSCs-EVs on polarization of macrophage, the mouse-derived macrophages were stimulated with various concentrations of hUCMSCs-EVs (0, 5, 10, 20, 40, and 80 μg/mL) for 2 days, after that, the culture medium was substituted with fresh medium without hUCMSCs-EVs, and the macrophages were continued to grow for another 2 days. Then the polarized macrophages were analyzed by PCR analysis and flow cytometry. Independent experiments were performed three times.

### Uptake of small extracellular vesicles derived from hUCMSCs

Uptake of hUCMSCs-EVs was analyzed through co-cultured macrophages with DiR-labeled hUCMSCs-EVs, as reported previously [48]. Briefly, DiR-labeled hUCMSCs-EVs were used and co-incubated with the macrophages at 37 °C for 6 h. Subsequently, these macrophages were washed with sterile PBS to remove uninternalized hUCMSCs-EVs, and the DiR-labeled hUCMSCs-EVs (red dots) were observed by confocal microscope (ACEA NovoCyte).

### Induction of OA chondrocytes by IL-1β and treatment with hUCMSCs-EVs in vitro

The rat-derived chondrocytes were placed in Petri dishes with DMEM/Basic, including 10% FBS (Gemini) and penicillin–streptomycin mixture (1%), followed by grown under standard incubational environment (37 °C, 5% CO_2_). Then the stimulation of chondrocytes with 10 ng of IL-1β was carried out. Subsequently, the OA chondrocytes caused by IL-1β were treated with a different solution. The experiment was divided into three groups according to the addition of reagents: the PBS group (negative control): OA chondrocytes were treated with PBS; the supernatant of M2 macrophages (M2S) group: OA chondrocytes were treated with the supernatant of M2 macrophages induced by hUCMSCs-EVs; the platelet-rich plasma (PRP) group: OA chondrocytes were treated with 10 ng/mL of PRP.

### Cell counting kit-8 analysis

A suitable amount of reagent from cell counting kit-8 (10 μL) (Biosharp, BS350B) was added into the cells that were plated in the 96-well plate (1 × 10^3^ cells each well) at 1 day, and then the cells were further cultured for three hours. And the optical density (OD450 nm) values were analyzed by utilizing a microplate reader.

### Histology staining and immunohistochemical analysis

The repaired cartilage tissues were collected and subjected to histopathological examination. The collected specimens were fixed with paraformaldehyde and then decalcified with 10% PBS-buffered EDTA for 35 days. The decalcified samples were fixed and embedded using paraformaldehyde and paraffin wax, respectively. After that, the cartilage was sectioned into 5 μm, and hematoxylin and eosin (HE) and safranin O staining were carried out for these sections. The therapeutic effect of OA cartilage tissue was assessed according to the Osteoarthritis Research Society International (OARSI) scoring system [49]. The histological score of these specimens was calculated by two pathologists who were blind to the experiments. For the immunohistochemical analysis, 5 μm thick sections of cartilage tissue were treated with primary antibodies against ARG1 (Bioss, bs-23837R), CD206 (Bioss, bs-21473R) or MMP13 (Proteintech, 18165-1-AP), and observed by streptavidin peroxidase detection system based on the protocols after incubation with secondary antibody (Sangon Biotech, Shanghai, China).

### Flow cytometry

The cultured hUCMSCs were washed with PBS; then, 1 × 10^6^ hUCMSCs were placed in a centrifuge tube and treated with Human MSC Analysis Kit (562245, BD, USA). After 30 min of maintenance in the dark at 4 °C, hUCMSCs were washed twice by PBS and then resuspended in PBS. These marker proteins were detected by flow cytometry (BD Bioscience, BD FACSCalibur). The same method was used in the measurement of the polarization of M2 macrophages. For detection of specific markers, the suspended macrophages were incubated with antibodies, anti-CD63 (53-5920-80, eBoscience, San Diego, United States) and anti-CD81 (17-2061-82, eBoscience, San Diego, United States) at 4 °C kept in the dark place for 30 min to test the proportion of macrophages.

### RT-PCR assay

Total RNA from the culture cells was purified using a Total RNA kit (Magen, Guangzhou, China) following the instruction. Subsequently, the first-strand cDNA synthesis kit (Takara, Beijing, China) was applied to produce cDNA from RNA. The target gene transcripts were detected using SYBER green quantitative real-time polymerase chain reaction (PCR) SuperMix Plus (Roche, 50837000) through a real-time PCR system (Roche Company, Basel, Switzerland) based on the manufacturer’s protocols. The glyceraldehyde 3-phosphate dehydrogenase (GAPDH) was employed as the internal control. The procedure was set as 40 cycles of 95 °C for 15 s, 60 °C for 30 s, and followed by 72 °C for 30 s. Moreover, the relative expression of target genes was measured by the 2^–ΔΔ^CT method. The sequences of primer applied in this research are listed in Table 1.

**Table 1:** The PCR primers utilized in this study

Gene	Sense (5′–3′)	Antisense (5′–3′)
GADPG-M	ACTTGAAGGGTGGAGCCAAA	GCCCTTCCACAATGCCAAAG
ARG1-M	CATATCTGCCAAGGACATCG	GGTCTCTTCCATCACTTTGC
CD206-M	AGGGTGCGGTACACTAACTG	TCTGACTCTGGACACTTGCC
INOS-M	GGAGCGAGTTGTGGATTGTC	CAGCCTCTTGTCTTTGACCC
CD86-M	CTCAGATGCTGTTTCCGTGG	CTGTGCCCAAATAGTGCTCG
IL-1-M	ATGATGGCTTATTACAGTGGCAA	GTCGGAGATTCGTAGCTGGA
IL-10-M	GAGAAGCATGGCCCAGAAATC	GAGAAATCGATGACAGCGCC
IL-6-M	ATAGTCCTTCCTACCCCAATTTCC	GATGAATTGGATGGTCTTGGTCC
GAPDH-R	TCCAGTATGACTCTACCCACG	CACGACATACTCAGCACCAG
TNF-α-R	GATCGGTCCCAACAAGGAGG	GCTTGGTGGTTTGCTACGAC
MMP13-R	ACCATCCTGTGACTCTTGCG	TTCACCCACATCAGGCACTC
SOX9-R	TCCAGCAAGAACAAGCCACA	CGAAGGGTCTCTTCTCGCTC
ACAN-R	GAATGGGAGCCAGCCTACAC	GAGAGGCAGAGGGACTTTCG

M indicates mouse; R indicates rat

### Western blot assay

The RIPA lysis buffer (Solarbio, Beijing, China) was used to conduct the purification of the protein of the cells and hUCMSCs-EVs. The concentrations of extracted protein were tested and calculated by a bicinchoninic acid protein quantification kit (Thermo Scientific, 23225). A 40 μg of denatured protein was subjected to 10% SDS polyacrylamide gel, subsequently transferred onto the polyvinylidene fluoride membranes (Millipore Corp, Billerica, United States). And then these polyvinylidene fluoride membranes were incubated with first antibodies, anti-IL-4 (Boster, 10K274), anti-TNF-α (Proteintech, 17590-1-AP), anti-MMP13 (Proteintech, 18165-1-AP), anti-CD63 (Bioss, bs-1523R), anti-TSG101 (Abclonal, A1692), anti-CD81 (Bioss, bs-6934R), anti-CALNXIN (Proteintech, 10427-2-AP), or anti-GAPDH (Sangon Biotech Co., Ltd, China) at 4 °C for 12 h after blocking with 5% milk. The secondary antibody, anti-rabbit IgG (Sangon Biotech Co., Ltd, Shanghai, China), was used at 37 °C for one hour. The expression level of each protein was assessed by the Odyssey CLx imaging systems (Li-COR Biosciences, Lincoln, United States).

### High-throughput sequencing of miRNAs

The RNA of hUCMSCs-EVs was extracted by the MagZol (Magen) following the manufacturer’s instruction. The RNAs quantity was confirmed by the Qubit^®^2.0 (Invitrogen, United States), and their integrity was measured by Agilent 2200 T apeStation (Agilent T echnologies, United States). And then, the RNAs were firstly ligated with 3′ RNA adapter, followed by ligated with 5′ adapter ligation. Next, the RT-PCR was used to amplify the adapter-ligated RNAs in a low-cycle manner. Following the directions of NEBNext^®^ Multiplex Small RNA Library Prep Set for Illumina^®^ (Illumina, USA), the PAGE gel was used to size-select the PCR products. And then, Agilent 2200 T apeStation was applied to assess the purified library products. Subsequently, the libraries were subjected to sequencing by the HiSeq 2500(Illumina, USA) with single-end 50 bp at Ribobio Co. Ltd (Ribobio, China). And the expression of miRNAs was estimated by the Reads Per Million (RPM) values, in which the PRM is equal to (number of reads mapping to miRNA/ number of reads in Clean data) × 10^6^. Subsequently, the top five expressed miRNAs in hUCMSCs-EVs were analyzed, in which the bar graphs and percentage pie charts of the top five expressed miRNAs were plotted. Four prediction tools for target gene, TargetScan (http://www.targetscan.org), miRWalk (zmf.umm.uni-heidelberg.de/apps/zmf/mirwalk2/index.html), RNAhybrid (bibiserv.cebitec.uni-bielefeld.de/rnahybrid), and miRanda (http://www.microrna.org), were applied to forecast the target genes of selected five miRNAs. Gene Ontology (GO) and Kyoto Encyclopedia of Genes and Genomes (KEGG) pathway for these target genes were analyzed using clusterProfiler23 package in R.

### LC–MS/MS analysis

The tryptic peptides were dissolved in aqueous solution containing 0.1% formic acid and 2% acetonitrile. The gradient was comprised of an increase from 4% to16% solvent B (0.1% formic acid in 90% acetonitrile) over 38 min, 16% to 30% in 8 min and climbing to 80% in 4 min then holding at 80% for the last 4 min, all at a constant flow rate of 500 nL/min on an EASY-nLC 1000 UPLC system. The peptides were subjected to NSI source followed by tandem mass spectrometry (MS/MS) in Q ExactiveTM Plus (Thermo) coupled online to the UPLC. The electrospray voltage applied was 2.2 kV. The m/z scan range was 350 to 1600 for full scan, and intact peptides were detected in the Orbitrap at a resolution of 70,000. Peptides were then selected for MS/MS using NCE setting as 28 and the fragments were detected in the Orbitrap at a resolution of 17,500. A data-dependent procedure that alternated between one MS scan followed by 20 MS/MS scans with 15.0 s dynamic exclusion. Automatic gain control (AGC) was set at 5E4. Fixed first mass was set as 100 m/z.

### Database search

The resulting MS/MS data were processed using Proteome Discoverer (V2.4.1.15). Tandem mass spectra were searched against Homo_sapiens_9606_SP_20211214.fasta, and the reverse decoy database was added to calculate the false positive rate (FDR) caused by random matching. Trypsin/P was specified as cleavage enzyme allowing up to 2 missing cleavages. The mass tolerance for precursor ions was set as 20 ppm in First search and 5 ppm in Main search, and the mass tolerance for secondary fragment ions was set as 20 PPM. Carbamidomethyl on Cys was specified as fixed modification and oxidation on Met was specified as variable modifications. FDR was adjusted to < 1% and minimum score for peptides was set > 40.

### Experimental animals

All the animals used in the experiment were purchased from the Medical Laboratory Animal Center, Guangxi Medical University. All the animal operations involved in this study were carried out as prescribed by the Guide for the Care and Use of Laboratory Animals published by the National Institutes of Health (Eighth Edition) and were approved by the Animal Ethic Committee of the Guangxi Medical University. A total of 40 Sprague–Dawley (SD) rats (male, 6–8 weeks old, weighing about 250–300 g) were housed in standard conditions with 22 ± 1 °C of temperature and 65–70% of humidity. And every five rats were fed in one cage with access to water and food freely. The surgical procedures in the rat model were conducted as follows. After successful anesthesia with 2% pentobarbital sodium, the left knees of the rats were treated in a surgically sterile manner. For the OA animal model, the anterior cruciate ligament was transected with a scalpel after incision of the knee capsule. The prophylactic antibiotic treatment with thirty thousand units of penicillin (Hebei YuanZheng Pharmaceutical co., LTD) every day was given for 3 days after the operation. After the establishment of the OA model, the knee joints of rats were treated with different reagents and were separated into three groups as follows: PBS group: intra-articular injection with PBS; PRP group: intra-articular injection with PRP; hUCMSCs-EVs group: intra-articular injection with hUCMSCs-EVs. The rats in each group were injected with corresponding reagents once every week. And the repaired cartilage of the rats was collected at 4 weeks or 8 weeks for further investigation.

### Statistical analysis

All data gained in this research are presented as mean ± standard deviation (SD). The data were analyzed by GraphPad Prism 8.0 (San Diego, CA, USA). Unpaired Student’s t-test was applied to compare the difference between the two groups. The one-way analysis of variance was employed to compare multiple groups, followed by Tukey’s multiple comparison tests. The significant difference is indicated by a p-value < 0.05.

## Supplementary Information

**Additional file 1: Fig. S1.** HUCMSCs-EVs significantly promote the M2 polarization of human macrophages, THP-1. THP-1 cells were cultured with hUCMSCs-EVs (80 ng/ml) for 3 days after 24 h of stimulation with PMA (50 ng/ml), and expression of M1 macrophage markers (A) and M2 macrophage markers (B) was detected by quantitative RT-PCR, the experiment was performed triplicate; *p < 0.05, **p < 0.01, ***p < 0.001.

**Additional file 2: Fig. S2.** Effect of HUCMSCs-EVs on chondrocyte activity. (A) The activity of chondrocytes was measured after 24 h of induction with hUCMSCs-EVs (80 ng/ml) by Cell Counting Kit-8. (B) The number of viable chondrocytes was shown by the fluorescence images after 24 h of induction with hUCMSCs-EVs (80 ng/ml); green represents live cells while red represents dead cells; Scale bar: 1 mm.

## Data Availability

The data supporting the findings of this study are available from the corresponding author upon reasonable request.
