# Supplementary figures and images for: Anti-inflammatory and immunomodulatory effects of the extracellular vesicles derived from human umbilical cord mesenchymal stem cells on osteoarthritis via M2 macrophages
Source: J Nanobiotechnology. 2022 Jan 20;20:38. doi: 10.1186/s12951-021-01236-1 (PMC8771624; doi:10.1186/s12951-021-01236-1)

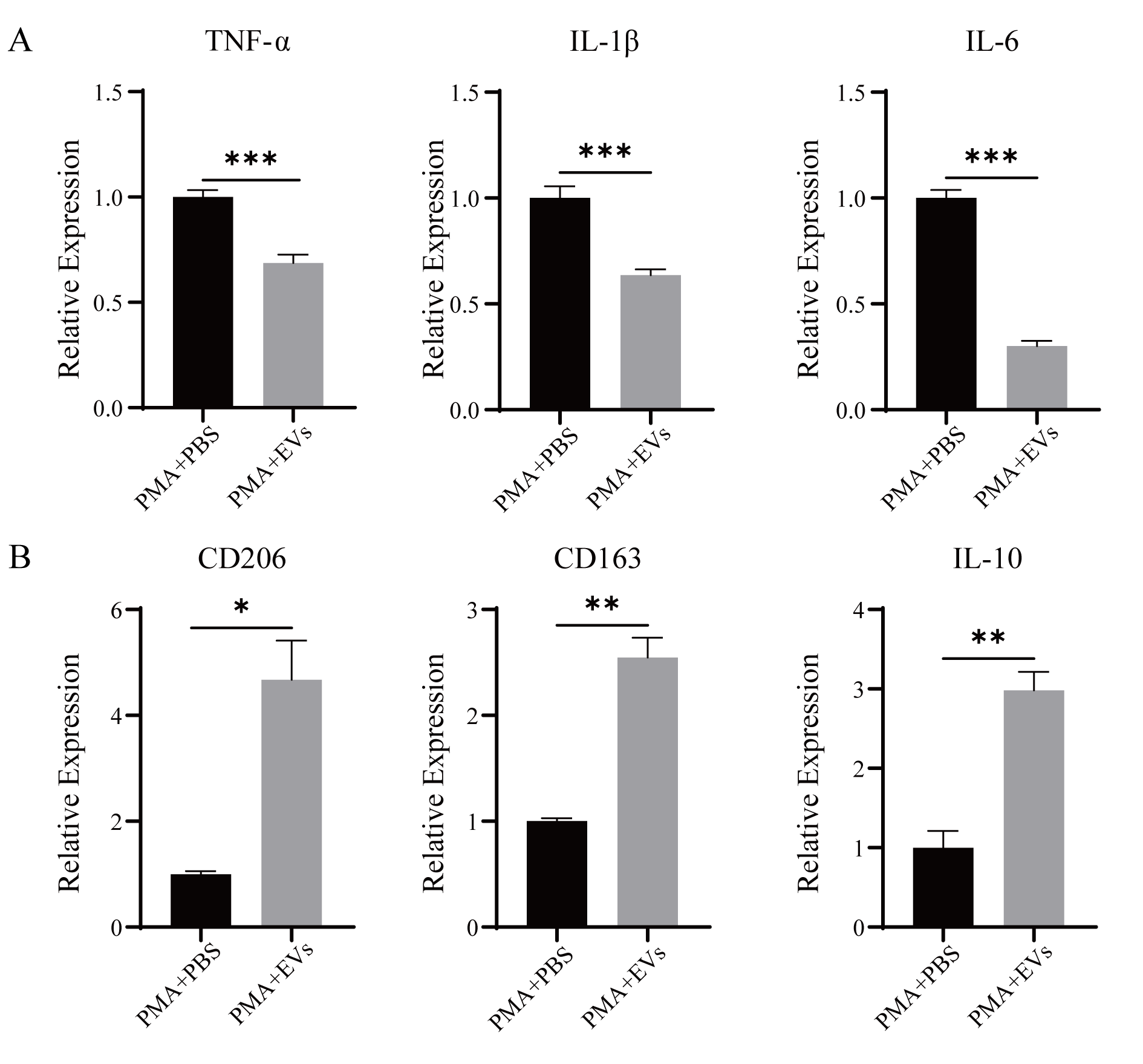

Supplement: Supplementary file 1 — Additional file 1: Fig. S1. HUCMSCs-EVs significantly promote the M2 polarization of human macrophages, THP-1. THP-1 cells were cultured with hUCMSCs-EVs (80 ng/ml) for 3 days after 24 h of stimulation with PMA (50 ng/ml), and expression of M1 macrophage markers (A) and M2 macrophage markers (B) was detected by quantitative RT-PCR, the experiment was performed triplicate; *p < 0.05, **p < 0.01, ***p < 0.001. [file 12951_2021_1236_MOESM1_ESM.tif]

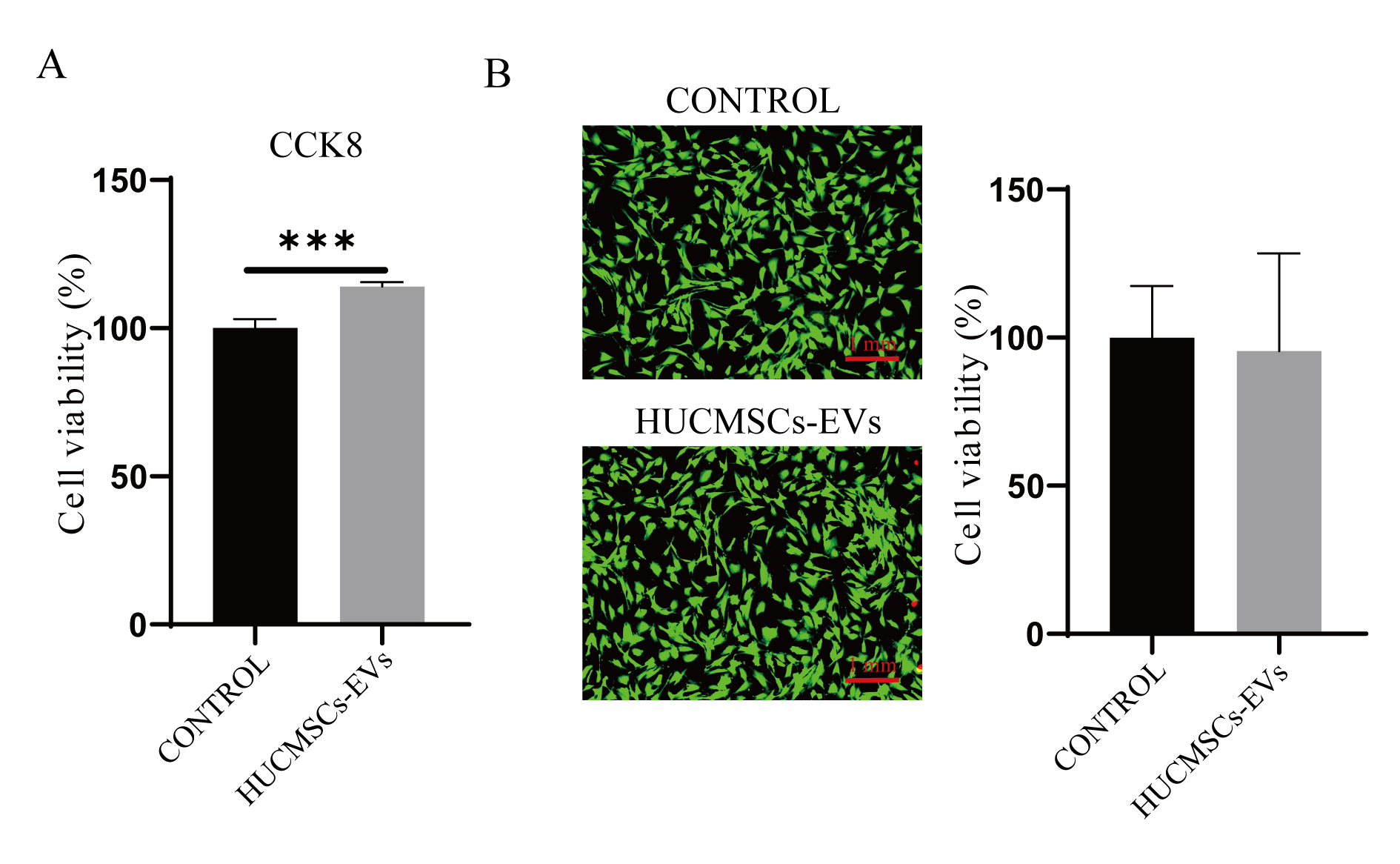

Supplement: Supplementary file 2 — Additional file 2: Fig. S2. Effect of HUCMSCs-EVs on chondrocyte activity. (A) The activity of chondrocytes was measured after 24 h of induction with hUCMSCs-EVs (80 ng/ml) by Cell Counting Kit-8. (B) The number of viable chondrocytes was shown by the fluorescence images after 24 h of induction with hUCMSCs-EVs (80 ng/ml); green represents live cells while red represents dead cells; Scale bar: 1 mm. [file 12951_2021_1236_MOESM2_ESM.tif]
